# Is the Immunological Response a Bottleneck for Cell Therapy in Neurodegenerative Diseases?

**DOI:** 10.3389/fncel.2020.00250

**Published:** 2020-08-11

**Authors:** Cristina Salado-Manzano, Unai Perpiña, Marco Straccia, Francisco J. Molina-Ruiz, Emanuele Cozzi, Anne E. Rosser, Josep M. Canals

**Affiliations:** ^1^Laboratory of Stem Cells and Regenerative Medicine, Department of Biomedicine, University of Barcelona, Barcelona, Spain; ^2^Production and Validation Center of Advanced Therapies (Creatio), Faculty of Medicine and Health Science, University of Barcelona, Barcelona, Spain; ^3^Institute of Neurosciences, University of Barcelona, Barcelona, Spain; ^4^Networked Biomedical Research Centre for Neurodegenerative Disorders (CIBERNED), Barcelona, Spain; ^5^August Pi i Sunyer Biomedical Research Institute (IDIBAPS), Barcelona, Spain; ^6^FRESCI by SCIENCE&STRATEGY SL, Barcelona, Spain; ^7^Department of Cardio-Thoracic, Vascular Sciences and Public Health, University of Padua, Padua, Italy; ^8^Transplant Immunology Unit, Padua University Hospital, Padua, Italy; ^9^Division of Psychological Medicine and Clinical Neurosciences, Cardiff University, Cardiff, United Kingdom; ^10^MRC Centre for Neuropsychiatric Genetics and Genomics, Cardiff University, Cardiff, United Kingdom; ^11^Brain Repair Group, School of Biosciences, Cardiff University, Cardiff, United Kingdom

**Keywords:** neurological disorders, regeneration, transplants, immune system, rejection

## Abstract

Neurodegenerative disorders such as Parkinson’s (PD) and Huntington’s disease (HD) are characterized by a selective detrimental impact on neurons in a specific brain area. Currently, these diseases have no cures, although some promising trials of therapies that may be able to slow the loss of brain cells are underway. Cell therapy is distinguished by its potential to replace cells to compensate for those lost to the degenerative process and has shown a great potential to replace degenerated neurons in animal models and in clinical trials in PD and HD patients. Fetal-derived neural progenitor cells, embryonic stem cells or induced pluripotent stem cells are the main cell sources that have been tested in cell therapy approaches. Furthermore, new strategies are emerging, such as the use of adult stem cells, encapsulated cell lines releasing trophic factors or cell-free products, containing an enriched secretome, which have shown beneficial preclinical outcomes. One of the major challenges for these potential new treatments is to overcome the host immune response to the transplanted cells. Immune rejection can cause significant alterations in transplanted and endogenous tissue and requires immunosuppressive drugs that may produce adverse effects. T-, B-lymphocytes and microglia have been recognized as the main effectors in striatal graft rejection. This review aims to summarize the preclinical and clinical studies of cell therapies in PD and HD. In addition, the precautions and strategies to ensure the highest quality of cell grafts, the lowest risk during transplantation and the reduction of a possible immune rejection will be outlined. Altogether, the wide-ranging possibilities of advanced therapy medicinal products (ATMPs) could make therapeutic treatment of these incurable diseases possible in the near future.

## Introduction

The term ‘neurodegenerative diseases’ refers to a heterogeneous group of disorders that affect the central or the peripheral nervous system with a wide array of clinical symptomatology, depending on the region that is affected. Neurodegenerative disorders are classified either by their clinical symptoms mainly motor, cognitive and psychiatric, by the proteins involved in the disorder (Ross and Poirier, 2004; Soto and Pritzkow, 2018; Agbas, 2019), or by the affected cell type, usually neurons or glial cells (Williams, 2002; Kovacs, 2014).

A wide range of neurodegenerative conditions have been recognized, with some of the best-known being Alzheimer’s, Parkinson’s (PD), Huntington’s (HD), and amyotrophic lateral sclerosis. PD and HD have well-characterized neuropathology and have been a focus of cell therapy research over the last three decades.

PD and HD present two main characteristics: the first is the progressive dysfunction of specific neurons, which initially occurs in a defined brain area; the second is worsening over time with eventual extension to involve additional cell types in more widespread brain areas (Williams, 2002; Hussain et al., 2018). Both are associated with aging and, in the majority of cases, present a life-threatening denouement.

## Parkinson’s Disease

PD is a progressive neurodegenerative disorder that presents a characteristic triad of motor symptoms: bradykinesia, tremor and rigidity. PD is thought to involve genetic and environmental factors, while the precise etiology is still unclear (Dickson, 2012). Despite current advances, there are no readily available specific biomarkers of PD, as may be available through genetic testing in monogenic disorders. Hence, a diagnosis of PD is normally based on clinical assessment of motor signs.

PD is associated with loss of dopamine (DA) neurons in the *Substantia Nigra pars compacta* (SNpc) (Fusco et al., 1999; Brichta and Greengard, 2014; Giguère et al., 2018), thus, at the anatomical pathology level, the two key hallmarks of PD are the selective loss of DA neurons of the SNpc, which results in a decrease of DA reaching the striatum, and the formation of intracytoplasmic α-synuclein (α-syn) protein aggregates known as Lewy Bodies (Spillantini et al., 1997; Sulzer and Surmeier, 2013).

The nigrostriatal DA pathway is the circuit primarily affected in PD, consequently making it the main target of the majority of cell-based strategies in this disease (Björklund and Stenevi, 1979; Perlow et al., 1979; Toledo-Aral et al., 2003).

**FIGURE 1:**
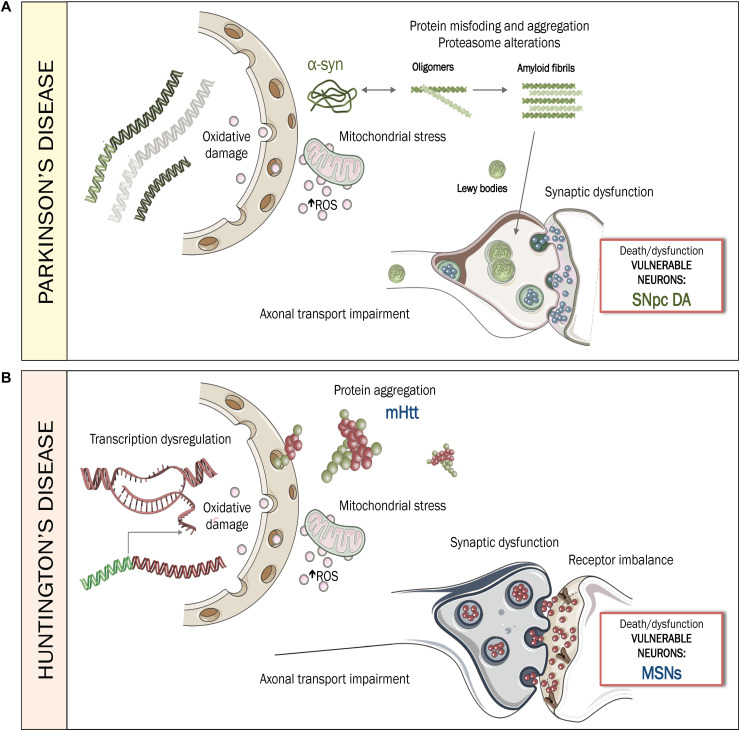
Pathogenesis of PD and HD. **(A)** Pathogenesis of PD, mediated by protein misfolding and aggregation of α-synuclein and the accumulation of intracytoplasmic Lewy bodies. Mitochondrial stress, augmentation of ROS and oxidative damage, together with axonal transport impairment and synaptic dysfunction, contribute to increase the vulnerability of SNpc DA neurons, leading to dysfunction or death during PD. **(B)** Pathogenesis of HD, mediated by aggregation of mHTT, transcriptional dysregulation, mitochondrial stress, augmentation of ROS and oxidative damage along with imbalances in axonal transport, synaptic connectivity and receptor regulation. Together, these disturbances contribute to increase vulnerability of MSNs, leading to dysfunction or death during HD. ROS: Reactive oxygen species. SNpc DA: substantia nigra pars compacta DAergic neurons. MSNs: medium spiny neurons.

The neuropathogenesis behind PD is still being elucidated. Here, some of the mechanisms underlying DA neuronal cell death are summarized (Figure 1A), based on neuropathological studies either from animal models or from human *postmortem* samples (Dauer and Przedborski, 2003; Hartmann, 2004). Several animal models of PD are available to study the disease (Blesa and Przedborski, 2014; Aron Badin et al., 2015), but none of them replicates human PD etiopathogenesis, nor accurately represents the anatomic organization of the human brain.

In-depth analyses of human *postmortem* samples have identified two key factor that compromise the viability of vulnerable neurons in PD (Figure 1A): proteostatic dysfunction, mediated by abnormal accumulation of misfolded proteins, such as α-syn and oxidative stress (Dias et al., 2013) which causes mitochondrial dysfunction, damage to nucleic acids and neuroinflammation (Blesa et al., 2015; Czarny et al., 2018; Guo et al., 2018). In addition, DNA integrity is compromised due to its intrinsic vulnerability to oxidative damage. Thus, the survival of affected neurons is uncertain, despite the compensatory efforts made by DNA-repair machinery (Gencer et al., 2012; Guo et al., 2018).

### Treatments for PD

**FIGURE 2:**
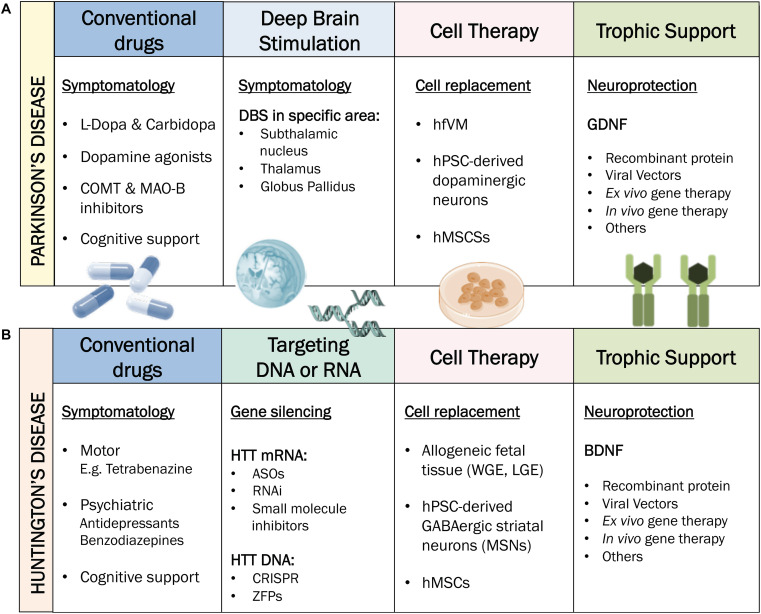
Current treatments for PD and HD. **(A)** Treatments for PD. Conventional drugs and DBS generally target motor symptoms of PD only and are usually accompanied by cognitive therapy. Cell therapy possesses disease-modifying potential through cell replacement. Trophic support is mainly seeking for a neuroprotective effect. **(B)** Treatments for HD. Conventional drugs are either targeting motor (e.g., tetrabenazine) or psychiatric symptoms (e.g., antidepressants or benzodiazepines). Therapies targeting DNA or RNA such as ASOs can be applied to silence HTT mRNA. Cell therapy possesses disease-modifying potential through cell replacement. Trophic support is mainly seeking for a neuroprotective effect. COMT: Catechol-O-methyltransferase. MAO-B: Monoamine oxidase B. DBS: Deep Brain Stimulation. hfVM: human fetal ventral mesencephalon. hPSCs: human pluripotent stem cells. hMSCs: human mesenchymal stem cells. GDFN: glial cell-line derived neurotrophic factor. ASOs: antisense-oligos. RNAi: RNA interference. WGE: whole ganglionic eminence. LGE: lateral ganglionic eminence. MSNs: medium spiny neurons. BDNF: brain-derived neurotrophic factor.

There are no established disease-modifying treatments able to slow, stop, or modify the disease course. Hence, at the moment available treatments only offer symptomatic relief of motor symptoms, with little clinical benefit in terms of the non-motor manifestations of PD (Figure 2A).

As the main hallmark in PD is the lack of DAergic innervation in the striatum, drug-based treatments rely on exogenous administration of compounds with DAergic activity (i.e., levodopa, DA agonists) to replace the depleted neurotransmitter (Zahoor et al., 2018). L-dopa is currently the most effective drug for PD. However, its long-term administration is linked to adverse effects such as dyskinesias and motor impairments. Fortunately, advanced treatments such as deep brain stimulation (DBS) have emerged as a complementary therapeutic approach for PD. DBS is an effective surgical intervention for PD, mediated by the application of chronic electrical currents to selected targets in the brain. The usual targets of DBS in PD are the subthalamic nucleus (STN) and/or the *globus pallidus internal* (*GPi*), depending on the dominant symptoms that need addressing (Krack et al., 2000; Negida et al., 2018).

The best clinical outcomes for PD have been achieved using DBS combined with pharmacological treatment (Wagle Shukla and Okun, 2014; Jakobs et al., 2019). DBS requires stereotactic surgery, so the procedure is associated with some risks and adverse effects. In some cases, worsening of cognitive, motor or psychiatric symptoms, and cerebral hemorrhages or stroke have been reported, while the potential for hardware failure must not be forgotten (Fang and Tolleson, 2017).

## Huntington’s Disease

Huntington’s Disease (HD) is an inherited neurodegenerative disease caused by a single mutation in the IT15 gene, known as huntingtin (HTT), that generates a toxic huntingtin protein (mHTT), leading to the dysfunction or death of medium spiny neurons (MSNs) from the striatum. HD is characterized by involuntary movements (chorea), cognitive and neuropsychiatric symptoms. Atrophy of the striatum (caudate and putamen nuclei), largely due to the loss of MSNs (Vonsattel et al., 1985), is one of the earliest and most striking changes in the brain in HD. However, many other brain areas are affected, such as cortex, *globus pallidus* or *thalamus* (Reiner et al., 1988; Kremer et al., 1990). The presence of the mutant HD gene can be detected through a highly reliable genetic test, usually performed on a blood sample, which also allows identification of gene positive, asymptomatic individuals. Disease onset most commonly occurs in mid-life and life expectancy ranges from around 15-30 years after disease onset, as there are no disease-modifying treatments currently available and symptomatic treatment is limited.

The neurotoxic (gain-of-function) properties of mHTT are probably accompanied by some loss of wtHTT properties (loss-of-function disease); both contributing to the pathogenesis of HD’s. Although a comprehensive understanding of the downstream cellular processes is still being sought, two processes that appear to play a key role in HD are altered protein homeostasis and disturbances in mitochondrial function (Figure 1B). Imbalances in the proteasome lead to the activation of proteases that cleave mHTT, generating more toxic species. In parallel, mitochondrial dysfunction is enhanced by defects in calcium homeostasis, aberrant ROS production and oxidative damages (Saudou and Humbert, 2016; Figure 1B). Brain-derived neurotrophic factor (BDNF) may also play a crucial role (Zuccato and Cattaneo, 2007; Baydyuk and Xu, 2012). BDNF is a potent neuroprotector with special affinity for striatal neurons and is decreased in HD due to the imbalances in transcriptional dysregulation and vesicular transport that also occur during the disease (Gauthier et al., 2004; Moumné et al., 2013). As previously described by our group, the decrease of BDNF induces dysfunction of enkephalinergic neurons which aggravates the symptomatology of HD (Canals et al., 2004). In fact, one of the most studied therapies preclinically has been the exogenous administration of BDNF, as it has demonstrated an ability to improve several motor and cognitive symptoms (Canals et al., 2004).

In conclusion, in HD, striatal MSNs receive a combination of pro-apoptotic signals in cell autonomous and non-cell autonomous ways that contribute to their vulnerability, leading to dysfunction or even death (Ehrlich, 2012; Morigaki and Goto, 2017).

### Treatments for HD

There is currently no neuroprotective, disease-modifying or curative treatment for HD available to clinical practice; only symptomatic treatments such as antidepressants, movement-suppressing drugs and physical therapy are available (Mestre and Ferreira, 2012; Figure 2B). Pharmacological relief of motor symptoms, such as chorea and dystonia, attempt to restore the balance of neurotransmitters including GABA, DA and glutamate (Van Vugt and Roos, 1999; Pidgeon and Rickards, 2013), but in general their efficacy is very limited.

To address the toxic gain-of-function of mHTT in HD, there is an extended field working on the development of huntingtin lowering therapies, with the most advanced currently being the lowering of mHTT levels by targeting its mRNA transcripts. Huntingtin-lowering strategies that target RNA or DNA (Figure 2B) are reviewed elsewhere (Carroll et al., 2011; Wild and Tabrizi, 2017; Tabrizi et al., 2019). RNA it is easily accessible in both nucleus and cytoplasm and is unprotected by repair machinery (Wild and Tabrizi, 2017). Therapies targeting RNA, such as antisense oligonucleotides (ASOs), aim to reduce the translation of HTT mRNA transcripts, which should theoretically inhibit all downstream toxic effects and slow, halt or reverse the progression of HD pathology and symptoms (Lane et al., 2018). Within RNA-targeting strategies, ASOs are the most advanced in the clinical pipeline, having already reached phase III clinical trials in HD patients under the name of Tominersen (previously known as RO7234292 and IONIS-HTT Rx; Ionis Pharmaceuticals, Inc.). The clinical trial (NCT03761849, 2018) is underway to evaluate the efficacy, safety, and biomarkers related to Tominersen compared to placebo in more than 800 early-stage HD patients, as part of GENERATION HD1 (Leavitt and Tabrizi, 2020). Although HTT-lowering therapies are promising, several questions remain open, such as the administration route, the potential toxicity due to lowering endogenous wild type, as well as mHTT by some ASOs, and the need to develop biomarkers that can report on the central lowering of mHTT and early reversal of neuronal dysfunction (Lu and Yang, 2012; Leavitt et al., 2020).

## Advanced Therapies to Treat Neurodegeneration

There are three types of Advanced Therapy Medicinal Products (ATMPs); gene therapy, cell therapy and tissue-engineered products. In addition, they can be combined with medical devices (so called combined ATMPs), for example, a biomaterial used to encapsulate a cell line that releases a neuronal pro-survival molecule (Lindvall and Wahlberg, 2008). In this review we will focus on the different strategies employed in cell therapy to treat neurodegeneration.

## Cell Therapy in Neurodegenerative Diseases

The ultimate goal of cell therapy in neurodegenerative diseases is to restore the lost function due to a neural circuit damage that occurs during neurodegeneration. To achieve this three main aspects have to be considered: (i) functional replacement of lost neural cells; (ii) enhancement of endogenous regeneration (which is a significant process in some regions of the adult brain in animal models, but its significance still widely discussed in human patients); and (iii) supply of pro-survival factors that are decreased because of the pathogenesis of the disease.

In this regard, two main strategies have been applied: human fetal neural tissue and stem cell-derived grafts. Among approaches using stem cells, two subgroups dominate: pluripotent stem cells (PSCs) and adult stem cells, such as mesenchymal stromal cells (MSCs). The first subgroup includes embryonic stem cells (ESCs) and induced pluripotent stem cells (iPSCs). When used in the context of cell therapy, PSCs are differentiated *in vitro* to the desired neural progenitor commitment is achieved prior transplant. In theory, at least, these cells represent an unlimited source of donor cells for transplantation. The second subgroup includes adult stem cells, such as MSCs, which are either delivered without major modifications or are reprogrammed *in vitro* towards a neuronal lineage. In some studies, MSCs have also been subjected to genetic engineering to enrich the therapeutic secretome that they cells naturally release (Dunnett and Rosser, 2004; Benraiss and Goldman, 2011; Barker et al., 2015a; Henchcliffe and Parmar, 2018).

Cell therapy approaches present many advantages over conventional treatments. Cell grafts can provide continuous replenishment of neurotransmitters, and hold the potential to integrate in the brain network and to produce long-term neuromodulation of the lost functionality in the basal ganglia (Piquet et al., 2012; Dunnett and Björklund, 2017). Either engineered to overexpress trophic factors or unmodified, stem cells release trophic factors such as BDNF and glial cell line-derived neurotrophic factor (GDNF) among others, as well as other growth factors and cytokines. The cocktail of small molecules continuously secreted by the cells has the potential to modulate neuroplasticity and induce neurogenesis (Drago et al., 2013; Salgado et al., 2015; Vogel et al., 2018; Reza-Zaldivar et al., 2019).

### Cell Therapy Strategies for PD

**FIGURE 3:**
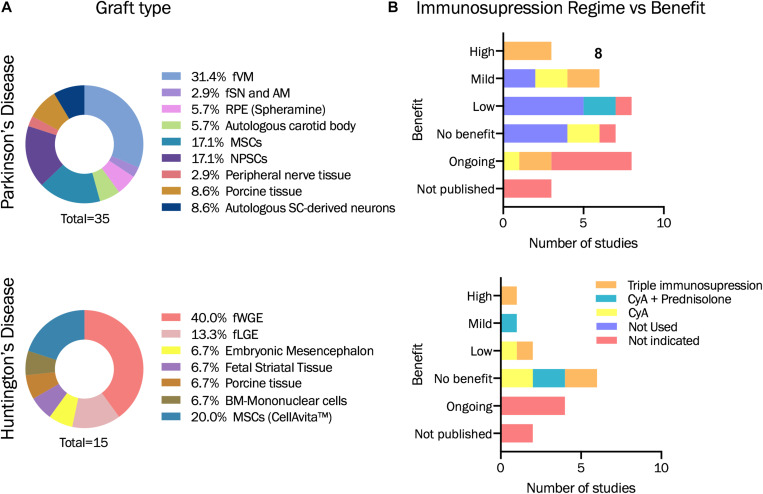
Descriptive statistics based on studies analyzed in Table 1. **(A)** Graft type used for each of the pilor study or clinical trial evaluated within this review for PD and HD. **(B)** Immunosupression regime administered and benefit obtained. fVM: fetal ventral mesencephalon. fSN and AM: Fetal *Substantia Nigra* and adrenal medula. RPE: retinal pigment epithelial cells. MSCs: Mesenchymal stromal cells. NPSCs: Neural progenitor or stem cells. SC-derived neurons: stem cell derived neurons. fWGE: fetal whole ganglionic eminence. fLGE: fetal lateral ganglionic eminence. BM: Bone-marrow. CyA: Cyclosporin A.

As described above, PD usually responds well to pharmacological medication in the short to medium term, but long-term DA repletion treatments led eventually to a variety of dopa-resistant motor complications, including difficult-to-treat motor fluctuations, dyskinesia, dystonia and freezing episodes, and non-motor signs including autonomic dysfunction, mood fluctuations and cognitive impairment (Fahn et al., 2004; Abbott, 2010). Driven by the need for physiological and localized delivery of DA, transplantation of fetal neural grafts led the way to diverse neuronal replacement strategies. Figure 3A summarizes cell-based therapy approaches administered in humans for PD and HD.

#### Allogeneic Fetal Neural Tissue

Autologous adrenal medullary tissue (AM) grafts were the first to be tested in patients with PD (Backlund et al., 1985; Lindvall et al., 1987; Madrazo et al., 1987; Drucker-Colín et al., 1988, 1999; Peterson et al., 1989; Hirsch et al., 1990; Goetz et al., 1991), but lacked sufficient supporting preclinical evidence and were associated with concerns about the efficacy (poor or absent functional outcome and poor survival of grafted cells) and safety (frequent complications from the surgery) of this approach, ultimately leading to its abandonment (Barker et al., 2015a). This failure was also partially blamed on the immune response generated from immunocompetent hosts (Piquet et al., 2012). Better outcomes were achieved using fetal ventral mesencephalic grafts (fVM), probably accounted for the better supporting preclinical data. The first published fVM implant, at the interface between the caudate nucleus and the lateral ventricle reported, was reported to have been associated with some improvement in PD symptoms although there was a lack of proper clinical assessment and long-term follow-up (Madrazo et al., 1988). The first fVM transplants into the striatum were associated with no improvement for the two PD patients of the study (Lindvall et al., 1989), but subsequent modifications of the grafting process resulted in graft survival in the caudate nucleus, DA release and improvement of motor symptoms of PD patients (Lindvall et al., 1990). These promising results led to the transplantation of 13 more patients in an open label study in Lund over the 1990s (Sawle et al., 1992; Lindvall et al., 1994; Wenning et al., 1997; Brundin et al., 2000). Despite an overall patient improvement and sustained benefits in some patients over more than 20 years (Kefalopoulou et al., 2014), efficacy was variable between individual participants (Piccini et al., 1999, 2000). Following the publications of these results, further groups in Europe, United States, and Canada applied a similar strategy in PD patients (Gildenberg et al., 1990; Freed et al., 1992; Widner et al., 1992; Kordower et al., 1995; Hauser et al., 1999; Freeman et al., 2000; Mendez et al., 2000, 2002). Unfortunately, although some results were promising, outcomes continued to be variable, ranging from clear benefits to poor or none (Boronat-García et al., 2017). The open-label studies were followed by two double-blind placebo-controlled trials. Although these trials were considered as a valuable continuation to previous studies, their design was significantly different to earlier open-label studies. In the first double-blind study bilaterally implanted grafts showed a modest recovery compared to the sham group (Freed et al., 2001), and the second double-blind study revealed a similarly variable symptomatic outcome (Olanow et al., 2003). Overall, these two trials did not provide significant improvements in patients with PD, especially when compared with other PD therapies such as DBS, although the primary outcome measures were highly subjective (Freed et al., 2011). In addition, these clinical trials reported some unpredictable and unacceptable side effects (i.e., dyskinesias) several years after the transplant in some of the patients (Allan et al., 2010) and older patients did not show any significant improvement (Krack et al., 2000). Retrospectively, it has been acknowledged that the design of both trials had many shortcomings, which resulted in them being underpowered and suboptimal. These limitations included the immunosuppressive regime, patient selection, cell preparation, fVM tissue handling and storage, surgical technique and graft location (Barker et al., 2013a). Furthermore, the original publications had relatively short follow-up periods, since it has been suggested that more time (3 to 5 years) would be required in order for the graft to produce significant clinical improvement (Ma et al., 2010). The modest improvement seen in some patients in the double-blind studies was associated with successful transplantation of DAergic cells that innervated the striatum and released DA, and as such confirmed the positive findings in the open-label studies which suggested that these factors were key for improvement of motor deficits. Efficacy of fetal transplant was further signaled by longer-term follow-up of some of the patients (Ma et al., 2010; Table 1).

**TABLE 1:** Summary of pilot studies and clinical trials in cell therapy for PD and HD.

Year	*n*	Graft	Administration route / Brain area	Assessment	Clinical benefits	Immunosuppression used	Graft survival analyzed?	Immune response	Clinical Trial Ref	References
***Parkinson’s Disease (PD): Open-label studies (1987-2000)***										
1987	1	fSN and AM	Unilateral stereotaxic implants. Caudate	UDPSR	Low	Ciclosporin A + Prednisone	No	Not evaluated	Early study (Mexico)	Madrazo et al., 1988
1987	2	fVM	Unilateral, CT-guided stereotaxic implants. Striatum	UDPSR, single-dose L-dopa tests, ^18^F-PET, D2 PET.	High	Triple immunotherapy (Cyclosporin A + Azathioprine +Prednisolone)	Yes (PET)	Not evaluated	Lund series	Lindvall et al., 1989, 1990, 1992, 1994; Piccini et al., 1999; Li et al., 2016b
1988	2	fVM	Unilateral stereotaxic implants. Putamen	^18^F-PET	No	Not indicated	Yes (PET)	Not evaluated	Early study (London, United Kingdom)	Sawle et al., 1992
1989	2	fVM	Bilateral, staged (2 weeks apart) CT-guided stereotaxic implants. Striatum	CAPIT: UPDRS, single-dose L-dopa tests, ^18^F-PET, cognitive tests.	Mild	Triple immunotherapy: Cyclosporin A (1 year) + Azathioprine (18 months) +Prednisolone	Yes (PET)	Not evaluated	Lund series	Widner et al., 1992
1989	6	fVM	Bilateral, staged (1-4 years) CT-guided stereotaxic implants. Striatum	CAPIT: UPDRS, single-dose L-dopa tests, ^18^F-PET, cognitive tests.	High	Triple immunotherapy (Cyclosporin A + Azathioprine +Prednisolone)	Yes (PET)	Not evaluated	Lund series	Wenning et al., 1997 Hagell et al., 1999 Kefalopoulou et al., 2014
1995	7	fVM	Bilateral, CT-guided stereotaxic implants. Striatum	UPDRS, ^18^F-PET, neuropsychological tests	Mild	IV methylprednisolone (surgery) + Cyclosporin A + Prednisolone	Yes (PET)	Not evaluated	Pilot study (Canada)	Freed et al., 1990, 1992
1995	6	fVM	Bilateral, staged (4 weeks) MRI-guided stereotaxic implants. Putamen	CAPIT: UPDRS, single-dose L-dopa tests, ^18^F-PET, cognitive tests.	Mild	Cyclosporin A (6 months)	Yes (PET, *postmortem* IHC)	Postmortem IHC: CD68 (microglia, macrophages), CD3 (T lymphocytes), L26 (B cells), HLA class II,	Pilot study (FL, United States)	Kordower et al., 1995, 1996, 1997, 1998, Freeman et al., 1995 Hauser et al., 1999
1995	12	Porcine fVM tissue	Unilateral MRI/CT-guided stereotaxic implants. Striatum	Safety, UPDRS, MRI, ^18^F-PET.	Low	Cyclosporin A (50% patients) // Graft treatment with monoclonal anti-MHC I Ab. (50% patients)	Yes (PET, *postmortem* IHC)	Postmortem IHC: CD3 (T lymphocytes), HLA class II.	Pilot study (MA, United States)	Deacon et al., 1997 Schumacher et al., 2000
1997	5	fVM	Bilateral, staged (0-6 months) CT/MRI-guided stereotaxic implants. Striatum	CAPIT: UPDRS, single-dose L-dopa tests, ^18^F-PET, H_2_^15^O PET, cognitive tests.	High	Triple immunotherapy (Cyclosporin A + Azathioprine +Prednisolone)	Yes (PET)	Not evaluated	Lund series	Brundin et al., 2000; Piccini et al., 2000; Kefalopoulou et al., 2014
2000	3	fVM	Bilateral, staged (4 weeks apart), MRI-guided stereotaxic implants. Putamen and *substantia nigra*	Safety, UPDRS, ^18^F-PET.	No	Cyclosporin A (6 months)	Yes (PET, *postmortem* IHC)	Postmortem IHC: CD45, CD68 (microglia, macrophages), GFAP (astrocytes)	Pilot study (Canada)	Mendez et al., 2002, 2005
***Parkinson’s Disease (PD): NIH studies, clinical trials using fVM and alternative cell sources (2001-2015)***										
2001	20	fVM	Bilateral, MRI-guided stereotaxic implants. Putamen	UPDRS, ^18^F-PET. Double-blind (control group).	No	Not used	Yes (PET, postmortem IHC)	Postmortem IHC: CD3 (lymphocytes), HLA class II.	NIH study (Canada) NCT00038116	Freed et al., 2001 Ma et al., 2010
2003	23	fVM	Bilateral, staged (1 week apart) stereotaxic implants. Putamen	UPDRS, ^18^F-PET. Double-blind (control group).	No	Cyclosporin A (6 months)	Yes (PET, postmortem IHC)	Postmortem IHC: CD45 (activated microglia, immune reactivity),	NIH study (United States)	Olanow et al., 2003
2003	6	hRPE cells linked to gelatin microcarriers (Spheramine)	Unilateral, MRI-guided stereotaxic implants. Putamen	Safety, UPDRS	Mild	Not used	No	Not evaluated	Pilot study	Watts et al., 2003 Bakay et al., 2004 Stover et al., 2005
2003	6	Autologous carotid body cells	Bilateral stereotaxic implants. Striatum	Safety, UPDRS	Low	Not used (autologous)	No	Not evaluated	Pilot study	Arjona et al., 2003
2004	1	Autologous hSC-derived neurons	Unilateral, MRI-guided stereotaxic implant.	CAPIT, UPDRS ^18^F-PET, MRI	Mild	Not used (autologous)	Unknown	Not evaluated	Pilot study	Neuman et al., 2009
2007	13	Autologous carotid body cells	Bilateral stereotaxic implants. Striatum	CAPIT, CAPSIT-PD. Long-term safety, UPDRS, ^18^F-PET	Low	Not used (autologous)	No	Not evaluated	Pilot study	Mínguez-Castellanos et al., 2007
2009	35	hRPE cells linked to gelatin microcarriers (Spheramine)	Bilateral, MRI-guided stereotaxic implants. Putamen	UPDRS. Double-blind (control group)	No	Not used	Yes (PET, postmortem IHC)	Postmortem IHC: CD19 (B cells), CD4 (natural killers, cytotoxic T cells), CD8 (helper T cells)	STEPS (NCT00206687)	Farag et al., 2009 Gross et al., 2011
2009	5	Autologous bone marrow stem cells	Stereotaxic implant. Striatum	UPDRS	No	Not used (autologous)	No	Not evaluated	NCT00976430 (Terminated)	
2011	20	Bone marrow MSCs	IV administration	Safety, UPDRS	Not published	Not indicated	No	Not evaluated	NCT01446614	
2013	4	NTCELL: immunoprotected (alginate-encapsulated) porcine choroid plexus cells. Xenograft	Intracranial stereotaxic insertion, guidance by neuroimaging	Safety, UPDRS, PET	Low	Not used	Not indicated	Not evaluated	NTCELL Phase I (NCT01734733)	
2013	15	Mesencephalic neural precursor cells	No data available	Safety, UPDRS, PET	Ongoing	Not indicated	Not indicated	Not evaluated	NCT01860794	
2014	8	Celavie human allogeneic undifferentiated NPCs from fetal brain tissue (OK99)	MRI-guided stereotaxic implant. Putamen	Safety, UPDRS, ^18^F-PET, MRI	Mild	Cyclosporine A (1 month)	Not specified	Flow cytometric analysis of antibodies against grafts and antibody-dependent cell-mediated cytotoxicity	HSCfPD (NCT02780895)	Madrazo et al., 2019
***Parkinson’s Disease (PD): TRANSEURO, MSC and NSC late clinical trials (2016-2020)***										
2015	16	Peripheral nerve tissue	DBS surgery. SN	Safety, UPDRS, MRI	Low	Not used (autologous)	Yes (MRI)	Not evaluated	NCT01833364	Van Horne et al., 2017, 2018
2015	20	fVM	Bilateral stereotaxic implants. Striatum.	UPDRS, ^18^F-PET. Double-blind (control group)	Ongoing	Triple immunotherapy (Cyclosporin A + Azathioprine +Prednisolone) for 12 months	Not indicated	Not evaluated	TRANSEURO (NCT01898390)	Barker et al., 2017, 2019
2015	12	hpNSCs (ISC-hpNSC^®^)	Bilateral MRI-guided stereotaxic implants. Striatum and SN.	Safety, UPDRS	No (ongoing)	Triple immunotherapy (Cyclosporin A + Azathioprine +Prednisolone)	Not indicated	Not evaluated	NCT02452723	Garitaonandia et al., 2016, 2018 Kern et al., 2018
2016	18	NTCELL: immunoprotected (alginate-encapsulated) porcine choroid plexus cells.	Intracranial stereotaxic insertion,	Safety, UPDRS	No	Not used	Not indicated	Not evaluated	NTCELL Phase II (NCT02683629)	Snow et al., 2019
2017	50	HLA-matched hESC-derived NPCs	MRI-guided stereotaxic implants. Striatum.	Safety, UPDRS, imaging.	Ongoing	Cyclosporin A	Not indicated	Not evaluated	NCT03119636	
2017	20	Bone marrow MSCs	IV administration	Safety, UPDRS, MRI, immune response changes	Low	Not indicated	Yes (MRI)	Measurement of plasma cytokines: inflammation (i.e., IL-6), cell growth and differentiation (i.e., BDNF) monocyte migration (MCP-1), and adaptive immune response (i.e., IL-12), HLA	NCT02611167	Schiess et al., 2019, 2020
2017	12	hNSCs	Nasal administration	Safety, UPDRS, MRI/PET, immunological index	Not published	Not indicated	Yes (MRI/PET)	Biomarker analysis: CD3, CD4, CD8, Treg cells	hNSCPD (NCT03128450)	
2017	12	Autologous MSCs	IV administration	UPDRS	Not published	Not indicated	No	Not evaluated	NCT04146519	
2018	20	Umbilical cord MSCs	IV administration	Safety, UPDRS	Ongoing	Not indicated	No	Not evaluated	NCT03550183	
2018	10	Umbilical cord MSC-derived NSCs	Intrathecal and IV administration	Safety, blood based biomarkers, CSF-based biomarkers,	Ongoing	Not indicated	No	Measurement of peripheral blood pro-inflammatory markers	NCT03684122	
2019	10	Autologous iPSC-derived NSCs	Not specified	Safety	Ongoing	Not specified	No	Not evaluated	NCT03815071	
2020	12	Stem cell-derived NPCs	Stereotactic delivery of cell suspension. Basal ganglia structures	UPDRS	Ongoing	Not specified	No	Not evaluated	NCT03309514	
2020	1	Autologous iPSC-derived DA progenitor cells	Bilateral, staged (6 months apart) MRI-guided stereotaxic implants. Putamen.	^18^F-DOPA PET, MDS-UPDRS, Hoehn & Yahr, MoCA, BAI, BDI, QUIP-RS, NMSS, PDQ-39	Low (Ongoing)	Not used (autologous)	Yes (MRI, PET)	Not evaluated (Ongoing)	Early Study (MA, United States)	Schweitzer et al., 2020
***Huntington’s Disease (HD): Pilot studies (1990-2008)***										
1990	4	Embryonic mesencephalon (pieces)	Bilateral CT-guided stereotactic implants. Caudate.	No formal clinical assessment	No	Cyclosporin A	No	Not evaluated	Early study (Cuba, Slovakia)	Šramka et al., 1992
1990	2	WGE (pieces)	Unilateral open microsurgery. Caudate.	No formal clinical assessment.	No	Cyclosporin A+ Prednisolone (6 months)	No	Not evaluated	Early study (Mexico)	Madrazo et al., 1993, 1995
1995	12	Porcine fVM tissue	Unilateral MRI/CT-guided stereotaxic implants. Striatum	Safety, UPDRS, MRI, ^18^F-PET.	No	Cyclosporin A (50% patients) // Graft treatment with monoclonal anti-MHC I Ab. (50% patients)	Yes (PET, *postmortem* IHC)	Postmortem IHC: CD3 (T lymphocytes), HLA class II.	Pilot study (MA, United States)	Fink et al., 2000
1995	14	LGE (pieces)	Bilateral MRI-guided stereotaxic implants. Striatum.	CAPIT-HD: UHDRS, neuropsychological tests, MRI, FDG-PET	No	Cyclosporin A (18-35 months.	Yes (MRI/PET, *postmortem* IHC)	Not evaluated	Pilot study (Los Angeles, CA, United States)	Philpott et al., 1997 Kopyov et al., 1998 Ross et al., 1999 Keene et al., 2007, 2009
1997	5	WGE (pieces)	Bilateral, staged (1 year apart) MRI-guided stereotaxic implants. Striatum.	CAPIT-HD: UHDRS, neuropsychological tests, electrophysiological tests, MRI, FDG-PET. Comparison with reference group.	High	Triple immunotherapy: Cyclosporin A (at least 6 months) + Prednisolone (1 year) + Azathioprine (1 year).	Yes (MRI/PET)	Not evaluated	Pilot study (Créteil, France)	Bachoud-Lévi et al., 2000a, c, 2006 Gaura et al., 2004
1998	7	LGE (pieces)	Bilateral, staged (1 month apart) MRI-guided stereotaxic implants. Striatum	CAPIT-HD: UHDRS, neuropsychological tests, MRI, D1, D2 and FDG-PET.	Low	Cyclosporin A (up to 6 months).	Yes (MRI/PET, *postmortem* IHC)	Postmortem IHC: GFAP (astrocytes), CD4 (T helper cells), CD8 (natural killers and cytotoxic T cells) HLA-DR (MHC-II)	Pilot study (FL, United States)	Freeman et al., 2000 Hauser et al., 2002b Furtado et al., 2005 Cicchetti et al., 2009, 2014 Cisbani et al., 2013
2000	5	WGE (suspension)	Unilateral MRI-guided stereotaxic implants. Striatum.	CAPIT-HD: UHDRS, neuropsychological tests, MRI, D2 PET.	No	Triple immunotherapy: Cyclosporin A + Azathioprine + Prednisolone (at least 6 months)	Yes (MRI/PET)	Inflammatory markers (C reactive protein)	NEST-UK pilot study (ISRCTN36485475)	Rosser et al., 2002 Barker et al., 2013b
2006	16	WGE (suspension)	Bilateral, staged (2-3 months apart) stereotaxic implants. Striatum	CAPIT-HD: UHDRS, neuropsychological tests, MRI, FDG PET, ^123^IBZM-SPECT. Comparison with reference group.	No	Oral methylprednisolone (2 weeks) + Azathioprine + Cyclosporin A (1 year)	Yes (MRI/PET)	Donor-specific HLA-antibody measurement	Pilot study (Florence, Italy)	Gallina et al., 2008a, b, 2010, 2014 Mascalchi et al., 2014 Paganini et al., 2014 Porfirio et al., 2015
2008	2	WGE (suspension)	Bilateral, staged (2-3 month apart) MRI-guided stereotaxic implants. Striatum	UHDRS, neuropsychological tests, MRI, D2 PET. Comparison with reference group.	Mild	Cyclosporin A (1 year) + Prednisolone (1 month).	Yes (MRI/PET)	Not evaluated	Pilot study (London, United Kingdom)	Reuter et al., 2008
***Huntington’s Disease (HD) (Cont.): MIG-HD, clinical trials (2001-2020)***										
2001	22	WGE (pieces)	Bilateral, staged (1 apart) stereotaxic implants. Striatum	Primary: UHDRS. Secondary: neurologic, cognitive, neurophysiologic, psychiatric, MRI PET.	Low	Triple immunotherapy: Cyclosporin A + Azathioprine +Prednisolone (18 months)	Yes (MRI/PET, postmortem IHC)	Donor-specific HLA-antibody measurement + postmortem ICH: CD45 (lymphocytes and microglia), CD28 (macrophages and activated microglia), GFAP (astrocytes), CD4 (T helper cells), CD8 (natural killers and cytotoxic T cells).	MIG-HD (NCT00190450)	Krystkowiak et al., 2007 Capetian et al., 2009 Krebs et al., 2011
2013	50	Bone-marrow derived autologous mononuclear cells	Intrathecal administration	Cognitive and behavioral effects	Not published	Not indicated	No	Not evaluated	BMACHC (NCT01834053)	
2016	6	MSC (CellAvita^TM^)	Intravenous administration	Primary: Safety. Secondary: preliminary efficacy (UHDRS, CIBIS, MRI), inflammatory markers, immunological response, HDRS	Ongoing	Not indicated	No	CD4+ and CD8+ proliferation and inflammatory markers (IL4, IL6, IL10, TNFa) release	SAVE-DH Phase I (NCT02728115)	
2017	35	MSC (CellAvita^TM^)	Intravenous administration	Primary: UHDRS. Secondary: CIBIS, MRI, HDRS, BMI. Triple-blind	Ongoing	Not indicated	No	Not evaluated	ADORE-DH Phase II (NCT03252535)	
2018	30	Fetal striatal cells	Surgical implantation	Long-term safety, feasibility (MRI/PET)	Ongoing	Immunosuppression for 12 months	Yes (MRI/PET)	MRI/PET scans to assess the development of clinically significant inflammatory or immune reactions	TRIDENT (ISRCTN52651778)	
2020	35	MSC (CellAvita^TM^)	Intravenous administration	Primary: UHDRS. Secondary: CIBIS, MRI, HDRS, BMI	Ongoing	Not indicated	No	Not evaluated	ADORE-EXT Phase II/III (NCT04219241)	

Clinical cell transplantation in PD was not further pursued until recently, when fVM grafts for PD were again considered for a multi-center trial (NCT01898390, 2013) by the TRANSEURO consortium (Barker et al., 2019). The trial was planned after re-analysis of available clinical data on human fVM transplantation, during which various factors associated with positive outcomes were identified, including the provision of adequate immunotherapy (Barker et al., 2015a). TRANSEURO has been systematically and rigorously designed, having adopted well-defined criteria for a number of parameters including patient selection, tissue detection, preparation, grafting, trial design and, most importantly, immunosuppression (Barker et al., 2019). Results from the TRANSEURO trial are not expected before 2021.

#### Embryonic Stem Cells

In order to overcome the main drawbacks of fetal-derived tissue, human ESCs were proposed as an alternative cell source from which to derived DAergic neuronal progenitors. Following differentiation towards the specific neural or neuronal lineage, these cells have the potential to be an unlimited source of donor cells for transplantation.

Early differentiation protocols yielded few neurons positive for the limiting enzyme for DA synthesis, tyrosine hydroxylase (TH). Subsequent strategies specifically aimed to generate DA neurons (Kawasaki et al., 2000; Kim et al., 2002; Barker, 2014) yielded relatively high numbers of TH-positive DA cells, but did not show co-expression of some transcription factors specifi for midbrain DA neurons (e.g., FOX2A). Along with tumor formation *in vivo* in some cases, the overall outcomes of early studies in animal models were not positive (Park et al., 2005; Roy et al., 2006; Sonntag et al., 2007), but new approaches rapidly emerged and more recent hESCs differentiation protocols have resulted in much more reliable production of midbrain DA-producing neuroblasts which, following transplantation into adult rodent brains, showed survival in the absence of tumor formation or uncontrolled growth (Kriks et al., 2011; Kirkeby et al., 2012; Grealish et al., 2014; Steinbeck et al., 2015). In fact, hESC-derived midbrain DA neurons are currently being developed for application in clinical trials in the United States (NYSTEM-PD) and Europe (EUROPEAN STEM-PD) in the context of a global consortium, G-Force PD (Barker et al., 2015b, Barker et al., 2017; Fan et al., 2020). Australian (NCT02452723, 2015; Garitaonandia et al., 2016, 2018; Kern et al., 2018) and Chinese (Cyranoski, 2017; NCT03119636, 2017) groups have also started clinical trials involving hESC-based therapy for PD. In the case of the Australian trial, Kern et al. (2018) have reported successful grafts of cells from a human parthenogenetic-derived neural stem cell line in ten out of twelve planned PD patients, with eight of them having completed the one-year active phase and entered the 5-year safety follow-up phase. No data has been published yet for the Chinese study, which was launched in 2017, but is apparently still in the recruitment phase.

#### Induced Pluripotent Stem Cells

Human iPSCs (hiPSCs) are a source of patient-specific neurons, that can also produce DA neurons via similar protocols to the ones used for hESCs (Kikuchi et al., 2011, 2017). One of the key benefits of using hiPSCs is the possibility of autologous transplantation. Similarly to hESC-derived DA neurons, midbrain identity has been achieved in differentiated hiPSCs (Niclis et al., 2017; Tofoli et al., 2019). Transplants of hiPSC-derived DA progenitors in rodent and non-human primate models of PD have resulted in graft integration into existing neural networks with associated motor improvement (Hallett et al., 2015; Kikuchi et al., 2017; Niclis et al., 2017). Two clinical trials of iPSC-derived DAergic progenitors have been planned by members of G-Force PD; one using allogeneic iPSCs with up to 2 years of immunosuppression with FK506 (CiRA trial) whereas the other will use autologous iPSCs with no immunosuppression [summit for PD trial; (Barker et al., 2017; Fan et al., 2020)]. The first patient in the CiRA trial was transplanted with iPSC-derived DA neurons in 2018 at Kyoto University Hospital, although recruitment to this clinical trial has since been suspended (UMIN000033564, 2018).

#### Mesenchymal Stromal Cells

MSCs are multipotent cells that can generate osteocytes, adipocytes, and chondrocytes. They are usually obtained from bone marrow, umbilical cord or adipose tissue and expanded *in vitro* as adherent cells. MSCs have demonstrated certain degree of potential in differentiating into non-mesenchymal cell types, such as neurons. This finding was very promising as these cells can be easily obtained and used for autologous treatment in neurodegenerative diseases. The ability of MSCs to differentiate into dopaminergic neurons was demonstrated using specific *in vitro* conditions in rat MSCs (Guo et al., 2005; Wang et al., 2013; Welchko et al., 2018) as well as in human MSCs (Fu et al., 2006; Trzaska et al., 2007, 2009; Trzaska and Rameshwar, 2011; Singh et al., 2017; Khademizadeh et al., 2019).

A recent meta-analysis demonstrated that MSC transplants can exert beneficial effects in animal models of PD (Riecke et al., 2015). Indeed, direct striatal administration of MSCs, with or without prior differentiation, has resulted in improvement of motor function, protection of the nigrostriatal system, and improved striatal DA release in several studies using rodent models of PD (Kitada and Dezawa, 2012; Mendes Filho et al., 2018). Some studies have even reported reduced microglial activation and graft immunoreactivity, as well as enhancement of neurogenesis in the subventricular zone and neuroblast migration to the striatum (Staff et al., 2019). Immunohistochemical analyses have provided very little evidence of MSC differentiation into DA neurons upon transplantation (Khoo et al., 2011). Overall, the beneficial outcomes observed following transplantation of MSCs in animal models seem to be promoted by their immunomodulatory and neurotrophic activity, rather than their potential ability to differentiate into functional neurons, which is in fact very limited *in vivo* (Cova et al., 2010; Lescaudron et al., 2012; Teixeira et al., 2017). Genetically engineered MSCs overexpressing TH, VEGF, GDNF or CDNF have also been used in PD mouse models with mixed, although overall positive, results (Staff et al., 2019). Along these lines, MSC-based GDNF secreting cells exert neuroprotective effects in inflammation-driven rat models of PD (Hoban et al., 2015). It has been found that secreting GDNF not only has positive effect on the viability and neural-like cell differentiation capacity of hMSCs, but it could also promote the therapeutic effectiveness of this delivery in a PD mouse model, since GDNF overexpression increases the viability and proliferation of stem cells (Sun et al., 2020).

Small open-label studies have demonstrated short-term safety of MSC grafts from healthy donors although no clinical benefit has been demonstrated (Venkataramana et al., 2010, 2012). Several clinical trials worldwide are currently applying allogeneic MSCs intravenously to treat PD. At this early clinical phase, the outcome is most commonly measured on the basis of safety and tolerability of the procedure, although most of these studies the Unified Parkinson’s Disease Rating Scale (UPDRS) as an additional outcome measure (Díaz, 2019).

### Cell Therapy Strategies for HD

HD displays a number of biological features, which make it a good model to explore how stem cell therapy can replace lost neurons. The fact that the disease involves significant atrophy of the striatum, with a relatively focal area of degeneration and predominantly loss of a single cell type (MSNs), provides to be a target for cell replacement strategies (Dunnett and Rosser, 2004; Figure 3A).

#### Allogeneic Fetal Neural Tissue

Administration of allogeneic fetal neural tissue in the striatum, which started to be considered more than 30 years ago (Table 1), has provided the rationale for exploring cell replacement in HD patients. So far, the most convincing evidence supporting potential effectiveness of cell replacement strategies to treat HD comes from animal and human studies which use transplantation of donor cells derived from dissection of the whole ganglionic eminence (WGE) (Döbrössy and Dunnett, 2003; Mazzocchi-Jones et al., 2009; Pauly et al., 2012), which gives rise largely to striatal brain structures (Graybiel et al., 1989). A short time after the first early-study WGE transplants (Table 1), the strategy was modified to enrich the population of striatal precursor cells, by dissecting only the lateral ganglionic eminence (LGE), rich in MSNs. Some studies revealed some behavioral improvement in rodents using both strategies, but survival and striatal graft volume were greater in WGE grafts (Kordower et al., 1995). WGE is the area of the fetal brain which eventually becomes the adult striatum and where the MSNs develop (Deacon et al., 1994; Olsson et al., 1995, 1998; Marín et al., 2000; Evans et al., 2012; Straccia et al., 2016). Hence, cells from this area are considered the “gold standard” for cell replacement in HD as the donor cells have the capacity to differentiate into the target cell type (Precious et al., 2017). It is important to bear in mind that optimal grafts are those derived from fetal WGE collected during the peak period of MSN neurogenesis (around 8-10 weeks of gestation in humans) (Dunnett and Rosser, 2011), whose transplantation has been shown to reduce or delay motor and cognitive deficits in animal studies including rats and non-human primates (McLeod et al., 2013; Schackel et al., 2013; Paganini et al., 2014; Yhnell et al., 2016). These studies have demonstrated that implanted cells can create functional synaptic connections and integrate into the neural circuitry, provided they meet the following conditions**:** (i) they are obtained during the appropriate developmental window; and (ii) they are directed to a GABAergic MSN fate (Dunnett and Rosser, 2014).

Overall, clinical studies of striatal allografts in HD patients have not present major complications associated with the surgery, but there is not enough data supporting its long-term beneficial effects. In addition, there have been complications derived from the immunosuppressive treatment in some patients, such as renal impairment, anemia, wound infections, and allograft rejection (Wijeyekoon and Barker, 2011). Also, heterogeneity and shortage of fetal tissue of suitable quality for transplantation is an issue. Given the need for other more readily available sources that could give rise to striatal MSNs, ESCs, iPSCs and MSCs have also been investigated as donor cell sources for HD, mainly in preclinical studies in animal models.

#### Embryonic Stem Cells

hESCs need to be differentiated to MSN progenitors *in vitro* before transplantation, in order to achieve lineage specificity and reduce the risk of tumorigenesis. Aubry et al. (2008) described the differentiation of human ESCs into MSNs *in vitro*, and the subsequent transplantation into a rodent model of HD. Other studies have also reported MSN differentiation protocols and have shown that grafts of the differentiated progeny could survive and develop into neurons, although in some cases transplanted cells did not differentiate into MSN-like neurons (Joannides et al., 2007; Song et al., 2007; Vazey et al., 2010). Positive effects on motor function impairment in the absence of MSN generation has also been reported (Song et al., 2007), possibly due to expression of neuroprotective factors by the transplanted cells. However, most studies have reported the generation of MSNs following transplantation into the lesioned striatum, showing that transplanted neural progenitor cells (NPCs) can survive, differentiate and integrate into the host, extending fibers over a long distance including into the *SN and globus palidus*, natural striatal targets (Aubry et al., 2008; Delli Carri et al., 2013; Nicoleau et al., 2013; Arber et al., 2015; Faedo et al., 2017; Comella-Bolla et al., 2020). Earlier studies reported teratoma formation (Aubry et al., 2008), but more recent studies with optimized differentiation protocols have reported successful transplant survival with no evidence of tumor formation (Arber et al., 2015; Comella-Bolla et al., 2020).

#### Induced Pluripotent Stem Cells

MSNs can also be generated from hiPSCs *in vitro*. In fact, most of the protocols mentioned for hESCs were also designed to work for hiPSCs (An et al., 2012; Jeon et al., 2012). Human iPSC-derived neural stem cells (NSCs) from an HD patient demonstrated MSN differentiation and functional improvement in mouse models (Jeon et al., 2014). However, mHTT aggregates were detected 33 weeks post-transplant, which highlights the possibility that auto-transplantation of cells derived from HD patients that still carry the HD mutation will eventually lead to persistence of the HD phenotype and cell death (Jeon et al., 2012). In contract, correction of the mutation in an HD-patient derived iPSC line resulted in the corrected cells not only surviving after transplantation, but also showing successful differentiation into a MSN phenotype (An et al., 2012). Besides these positive reports, long-term transplant studies are essential before clinical application to better clarify the mechanisms underlying therapeutic effect following transplantation of hPSC-derived NSCs (Lescaudron et al., 2012).

#### Mesenchymal Stromal Cells

Efficacy of human bone marrow-derived hMSCs (hBM-MSCs) after transplantation into the striatum has been examined in different rodent models of HD. HBM-MSCs have survived following transplantation, although only a minority of cells expressed a GABAergic phenotype and reduced the number of apoptotic cells in the striatum in a transgenic (R62-J2) and in a quinolinic acid (QA)–lesioned model. Motor improvements were only seen following transplantation in the QA-lesioned model (Lin et al., 2011). Another study showed that hBM-MSCs implanted into a transgenic HD mouse model (N171-82Q) increased endogenous neurogenesis and decreased atrophy of the striatum (Snyder et al., 2010). In addition, transplantation of rat BM-MSCs also elicited motor improvements in QA-rats as well as an increase in striatal volume (Jiang et al., 2011). As was the case following MSC transplantation in PD, the improvements observed after transplantation of MSCs in the striatum of HD animal models are very likely related to the therapeutic secretome that the cells release (Drago et al., 2013; Teixeira et al., 2017). It has been proposed that MSCs act through various mechanisms, including induction of NPC proliferation, chemokine secretion to promote endogenous NPCs cell recruitment and enhancement of neuronal differentiation (Connor, 2018). MSCs could also be useful in reducing the immune response occurring as a part of HD (Dalrymple et al., 2007; Björkqvist et al., 2008; Wild et al., 2011). In accordance with these results, Kwan et al. (2012) reported a therapeutic benefit of intravenous delivery of bone marrow-derived MSCs for the treatment of HD through modification of immune cell dysfunction.

Another approach which has gained traction as a potential HD therapy recently is the use of MSCs engineered to stably overexpress BDNF (Annett et al., 2013; Fink et al., 2015; Deng et al., 2016). There is evidence that intrastriatal delivery of hMSCs overexpressing BDNF causes a significant reduction in anxiety-like behaviors, a reduction of striatal atrophy, a significant increase in neurogenesis and extended lifespan in immune suppressed HD mouse models (Pollock et al., 2016).

To date, MSCs have been demonstrated to be effective in some cases, and to be safe and feasible for treatment in HD. However, several steps need standardization before further clinical trials of hMSCs in neurodegeneration-affected patients. For instance, aspects such as cell source (i.e., bone-marrow, adipose-derived, umbilical cord) and passage, route of administration, localization and number of injections would need to be standardized according to the purpose of the study and based on robust preclinical data.

So far, the majority of clinical trials using hMSCs have chosen an intravenous route of administration (Table 1), relying on previously demonstrated safety and the ability of MSCS to cross the BBB and travel to injury sites (Ra et al., 2011; Lykhmus et al., 2019). To our knowledge, the number of functional cells reaching target areas (such as striatum) upon administration in HD patients has not been published.

The question of whether the use of hMSCs will lead to therapeutic benefits to HD patients can only be answered once standardized and properly-designed studies are performed and their outcomes are analyzed.

## Immunogenicity of Striatal Grafts

As the field of regenerative medicine grows and new advanced therapies arise, clinical practices need to be continually adapted and updated. The intervention strategy and the analysis of the outcomes are tailored according to the type of therapy being considered and can be highly complex when it comes to cell and gene therapies or tissue engineering approaches. The potential impact of cell therapy as a disease-modifying strategy that could replace damaged neurons in neurodegenerative diseases such as HD and PD is beyond doubt. Nevertheless, the successful application of this type of advanced therapy relies on multiple factors, and among these factors, the host immune response is of critical importance.

The original idea of the CNS as an absolute immune-privileged site was proven to be wrong, however, there is a certain degree of privilege (Barker and Widner, 2004; Louveau et al., 2015, 2016a,b). Several studies have shown an innate and an adaptive immune response to allogeneic and xenogeneic cell transplants in the CNS, compromising the survival and functionality of the grafts (Hoornaert et al., 2017). Nevertheless, the immune response upon transplantation in the CNS differs to the response of allo- and xenografts transplants in the periphery, which is much more vigorous and immediate.

### Critical Factors That Affect the Immune Response Upon Cell or Tissue Grafting

When designing any given cell therapy, the aim is to maximize the therapeutic benefit (functionality, survival and integration) causing minimal injury, in order to obtain a positive balance of risk and benefit for the patient. To achieve this, there are critical factors that could impact the immune response and should be taken into consideration when implanting cells into the CNS to treat neurodegeneration.

The first is related to the transplantation procedure. Despite new techniques being minimally invasive and extremely accurate, the delivery of any cell into the brain involves penetration of the brain by a surgical instrument, which inevitably disrupts the BBB, leading to reactive astrogliosis and microglia activation and opens up the opportunity for lymphocytes to enter the CNS. Immunosuppression is needed to overcome inflammation and morbidity associated with the transplant procedure, however, immunosuppression therapy can act as a double-edged sword, as it can cause toxicity and worsen the clinical scenario if the regime is not accurately selected and monitored (Graybiel et al., 1989; Olanow et al., 2003; Ideguchi et al., 2008).

The second factor is the graft itself: the cell type used (fetal tissue, ESCs, iPSCs, NPCs, MSCs), its genetic modifications (if any) and the degree of mismatch between donor and recipient. Within the latter, four categories can be delineated: discordant xenograft (divergent species), concordant xenograft (closely related species), allogeneic (genetically different, but derived from individuals from the same species), including syngeneic (genetically identical), and autologous (same individual) grafts. The immune response observed for each of the categories is different. Hoornaert et al. (2017) described the immune response against discordant xenografts transplanted into the brain as usually involving the adaptive system through a cascade of events involving antibodies, complement system, natural killer cells and T-cell mediated responses (Abbas et al., 2017). Concordant xenografts and allografts should theoretically induce the same response, this being mediated by T-cells as CD4+ and CD8+ infiltration into rejecting grafts, and activated microglia (Larsson and Widner, 2000; Barker and Widner, 2004). Lastly, autologous grafts have been shown to induce certain degree of inflammation at the grafted site, which should subside rapidly without precluding graft survival (Hoornaert et al., 2017).

Another important aspect is the compatibility of the major histocompatibility complex (MHC), known in humans as HLA, human leucocyte antigen. The probability of graft rejection increases with the degree of mismatch between donor and host, and it could range from no rejection to a lifelong need of immunosuppressive therapy. Perfect HLA compatibility is difficult to achieve for allogeneic transplantation, although understanding the interactions of the graft with the recipient’s immune system is surely worth considering to achieve successful cell therapy outcomes (Taylor et al., 2011; Morizane et al., 2017). Contrary to previous reports showing that matching HLA haplotypes would reduce the need for immunosuppression following transplantation, a recent study by Aron Badin et al. (2019) has provided evidence of an immune response even in the context of an MHC-matched allograft, suggesting that, even in such an immunological combination, immunosuppression may needed to obtain long-term graft survival.

Furthermore, the transplantation site itself also plays an important role in graft survival. Barker & Widner (Barker and Widner, 2004) described the putamen as a region supporting higher graft survival than other areas such as the hippocampus, although this could be due to non-immunological factors, such as the presence of trophic factors. Fainstein and Ben-Hur (2018) showed in a recent study that the hippocampus is an almost completely immune-privileged site that allows survival of NPC grafts, while the same grafts were immunorejected in the striatum (Fainstein and Ben-Hur, 2018). Further assessment of the effects of transplantation site using identical cell grafts and animal models is needed to accurately identify “tolerant” and “non-tolerant” CNS areas.

The last aspect to consider is of special importance, the neuroinflammation process observed in neurodegenerative disorders. Neuroinflammation occurs in HD and PD and is characterized by the activation of astrocytes, microglia and other immune-mediators, together affecting the integrity of grafted cells (Barker and Widner, 2004; Phatnani and Maniatis, 2015; Hickman et al., 2018; Stephenson et al., 2018). Thus, neuroinflammation can influence graft survival and integration, as well as its rejection.

In order to evaluate the effect of each of these factors, there is a need for standardization of immune-related aspects following cell transplantation. The accurate monitoring of the immunosuppressive treatment administered, their adverse effects and the analysis of neuroinflammatory biomarkers should be considered to be as relevant as other elements of a transplant trial, such as the accurate evaluation of clinical outcomes.

### Neuroimmunology in Health and Disease

The classic idea of the CNS as an immune privileged site, meaning the inability of this system to generate an immune response against an implanted graft has been reassessed. Several studies have demonstrated an immune response occurring in the CNS, although it is delayed compared to non-CNS reactions, due to the tight control exerted on the brain’s immune system through multiple signaling pathways (Carson et al., 2006). There are several factors that could explain this delay. From an anatomic point of view, the CNS is protected by the BBB, which acts as a dynamic barrier that separates nervous tissue from the peripheral environment. The BBB also maintains the optimal balance of chemicals to support the function of neurons and it is in charge of limiting the entrance of antibodies and activated immune cells into the CNS (Barker and Widner, 2004; Iwasaki, 2017). It used to be postulated that the brain lacked lymphocyte drainage so that only activated lymphocytes could cross the BBB (Weller et al., 2010). However, due to advanced imaging techniques, two different studies have recently shown the existence of functional lymphatic channels that drain molecules and immune cells from meninges and parenchyma into the cervical lymph nodes (Aspelund et al., 2015; Louveau et al., 2015).

A further major consideration is that the immunologic profile of the CNS is completely different to other tissues. Peripheral tissues have antigen-presenting cells (APCs), such as dendritic cells (DCs) that present antigens to T lymphocytes through the interaction of MHC and co-stimulatory molecules. This interaction leads to the activation of an adaptive immune responses (Schetters et al., 2018), but does not exist in the brain, at least in the same way. There are also other cell types in the brain, primarily glial cells, that play similar roles to APCs and act as an innate immune response barrier in the parenchyma. Astrocytes and microglia are glial cells with different developmental origins that reside in the CNS and collaborate in the innate immune response (Ransohoff and Brown, 2012). Astrocytes usually need activation by specific signals (Toll-like receptor, nod-like receptor, TLR and NLR respectively) to participate in the immune response upon injury or infection (Ransohoff and Brown, 2012). Astrocytes usually take part in modulating the immune response by secreting a wide array of molecules: neurotransmitters, cytokines and metabolic and trophic factors (Verkhratsky et al., 2016). In addition, microglia also contribute to neuroinflammation by secretion of immunomodulatory, pro- and anti-inflammatory molecules (Town et al., 2005).

Interestingly, neurodegenerative diseases also lead to microglial activation with uncertain outcomes (Pennell and Streit, 1997; Ransohoff and Brown, 2012). It is also important to emphasize that knowledge available about the immune profile of the CNS apply, to some extent, to diseased conditions such as PD and HD, as neurodegeneration can also be associated with immunopathologies such as neuroinflammation (Guzman-Martinez et al., 2019).

Chronic neuroinflammation is an important feature of both PD and HD (Stephenson et al., 2018; Guzman-Martinez et al., 2019). Whether it results from a reaction to the neuronal degeneration process or it is an acquired phenotype from dysfunctional immune cells, is currently unclear (Crotti and Glass, 2015). However, it is very likely that it is a combination of both. Neuroinflammation is regulated by mediators such as cytokines, chemokines or ROS, among other inflammatory molecules that are released by glial cells. In addition, neuroinflammation compromises the BBB permeability, which can increase the recruitment of peripheral immune cells, exacerbating the neuroinflammatory response. The extent of the disruption of BBB during neurodegeneration is still controversial (Cabezas et al., 2014).

### Neuroinflammation Biomarkers

The use of biomarkers allows access to information about a given tissue by measuring biological parameters (Table 2). In the case of neurodegenerative diseases such as PD and HD, there are specific biomarkers of neuroinflammation that help us to understand the human disease and that could also serve to monitor the recipient’s host response to a graft. Two key approaches have been used to monitor neuroinflammation in the brain; imaging methods such as positron emission tomography (PET) and quantification of specific neuroinflammatory markers, such as cytokines, by immunohistochemistry in *postmortem* samples. PET has been proposed as a method to evaluate microglial activation *in vivo*, as it it would allow longitudinal monitoring of astroglial signals in life. [(11)C]-PK11195 is one of the markers used in PET imaging of the brain to detect inflammation, and it correlates with microglial activation and the severity of striatal neuronal dysfunction in neurodegenerative diseases (Politis et al., 2015; Rocha et al., 2016).

**TABLE 2:** Summary of inflammatory biomarkers for PD and HD.

	PD	HD
Brain parenchyma	IL-6	IL-6
		IL-8
	TNF-α	TNF-α
	PET-Activated microglia	PET-Activated microglia
Cerebrospinal Fluid	CRP	PGLYRP2
	SAA	APOA4
	MCP-1	MMP-3
		MMP-9
	IFNγ	Clusterin
	IL-8	Complement factors
	IL-6	IL-6
	IL- α	TGF-β1
Peripheral blood or plasma	IL-1β	VEGF
	IL-2	MMP-9
	IL-10	Chemokines
	IL-17A	Eotaxin-3, MIP-1β
	MIF	MCP-1
	TNF-α	MCP-4
	Anti-HLA	Anti-HLA

Activated microglia have been detected *in vivo* by PET in basal ganglia and cortical regions of patients suffering with PD (Gerhard et al., 2006), and correspond to immunohistochemistry postmortem analyses that showed significant increase of TNF-α, and IL-6 in the putamen of PD patients (Sawada et al., 2006). In HD, immunohistochemistry studies of postmortem samples showed significant microglial activation accompanied by an increased number of astrocytes. Increased levels of proinflammatory cytokines, such as IL-6 and IL-8, have also been shown in HD patients’ striatae (Björkqvist et al., 2008). In addition PET studies have confirmed microglial activation in HD patients, even before symptoms onset (Rocha et al., 2016; Table 1).

Some studies have shown that the imbalance of cytokine profiles was also reflected in the CSF of neurodegenerative patients, for example the observation of an increase of proinflammatory cytokines (IL-6, IL-1β) in PD patients (Chen et al., 2018), as well as increased inflammatory mediators (PGLYRP2, APOA4), metalloproteinases (MMP-3 and MMP-9), clustering and complement factors in the CSF of HD patients (Rocha et al., 2016; Table 1). Therefore, the knowledge of the inflammatory and immunological profiles of the different neurodegenerative pathologies represents a valuable tool to understand the behavior of grafted cells, as the functionality of the graft could be affected by the presence of neuroinflammation (Barker and Widner, 2004; Ideguchi et al., 2008; Hoornaert et al., 2017).

Obtaining CSF samples is usually invasive, time consuming and carries a small risk of adverse events. For this reason, some groups have been focused on the search for peripheral biomarkers that can serve to monitor the state of the disease as well as the immune response caused by the graft. Peripheral blood analysis in PD has shown an increase of proinflammatory cytokines (Hu et al., 2017; Chen et al., 2018), indicating the presence of inflammation associated to neurodegeneration (Table 1). The pro-inflammatory cytokines IL-6, TGF-β1 as well as vascular growth factor (VEGF), matrix metallopeptidase 9 (MMP-9) and chemokines eotaxin-3, MIP-1β, MCP-1 and MCP-4 have also been reported to be increased in HD patients (Dalrymple et al., 2007; Chang et al., 2015; Connolly et al., 2016). Moreover, IL-6 levels in peripheral blood were correlated with stage of HD pathology as represented by the UHDRS scale (Wild et al., 2011). Interestingly, a peripheral proinflammatory process is evident even in the pre-manifest stages (Björkqvist et al., 2008).

### Neuroinflammation Markers: Assessment in Cell-Based Clinical Trials for PD and HD

The success of a cell transplant in the CNS is not only due to its ability to integrate into the CNS parenchyma but also to the functionality of the cell graft to restore the proper neural connections in damaged tissue. For this reason, in order to understand the cellular and immunological response of the graft, two crucial aspects must be addressed. First, preclinical studies must be carried out in models that recapitulate the inflammatory events of the pathology, in order to understand the response of the cellular graft. Second, it is important to include measurements of the inflammatory and immunological parameters as a primary or secondary outcome in clinical trials of cell transplantation. To date, many studies have analyzed the immune response following cell engraftment (Table 1). However, there is a lack of standardization of this assessment, thus reducing the opportunities to draw clear conclusions.

Several clinical trials based on the treatment of PD or HD with cell therapy products have already included inflammatory and immunological mediators as measures (Table 1).

The first report of immune response monitoring after striatal cell transplantation in PD comes from the publication of the *postmortem* histological analysis of porcine xenografts that were placed unilaterally into the striatum of a cyclosporin A (CyA)-immunosuppressed PD patient (who died 7.5 months post-transplantation). As reported by Deacon et al. (1997), absent or little cellular infiltration and inflammatory response to the xenogeneic cells was found, which was confirmed by a relative low reactivity of markers for human T-cells and microglia (CD3, MHC-II) confined to the proximity the pig graft. These observations do not preclude an immune response, but may indicate that any response that had been mounted was relatively weak (Deacon et al., 1997; Schumacher et al., 2000). However, it cannot be ruled out that a strong response was mounted early on and had resolved by the time the *postmortem* can undertaken. Kordower et al. (1997), also documented the *postmortem* analysis of two PD patients transplanted with human fVM, who died around 19 months post-transplantation. In this case, the immune response to the grafts was assessed by immunostaining of MHC-II and markers for T-cells (CD3), B-cells (L26) and macrophages and monocytes (CD68). MHC-II upregulation and immune cell reactions from a central origin, as microglia, and a peripheral origin such as B-cells, T-cells, and macrophages, were found within the nigral graft, with no associated significant adverse effects having been reported. However, the possibility that the presence of these immune cells could have attenuated neurite outgrowth and the clinical benefits of the transplants was contemplated by the authors (Kordower et al., 1997). Two other examples of clinical studies which incorporated monitoring of immune response after striatal transplantation of fVM tissue in PD patients are the double-blind NIH-funded trials, both of which failed to show significant clinical benefit (Freed et al., 2001; Olanow et al., 2003). In the Canadian study, which did not include immunosuppression, two post-mortem cases were analyzed. CD3 and MHC-II staining revealed some inflammatory cells in the transplant tracks and perivascular areas, which did not seem to be correlated with graft survival (Freed et al., 2001). The United States trial analyzed five *postmortem* cases by immunostaining, finding upregulation of CD45, as a marker of activated microglia in the grafted striatum and especially around graft deposits, which is consistent with an immune reaction. Once again, it was hypothesized that following discontinuation of immunosuppression with CyA (which only lasted for six months) this immune reaction could have limited the clinical benefits of the intervention (Olanow et al., 2003). These observations contrast with reports from Mendez et al. (2005), in which *postmortem* analysis of microglial CD45 and CD68 markers undertaken 3-4 years after surgery in patients, immunosuppressed for only 6 months post operatively, revealed that the transplants were only mildly immunogenic to the host brain in the *SN* and the putamen. There were no major microglial reaction in the host tissue, and most CD45 or CD68 positive microglial cells showed resting morphology with only a mild reaction around the needle tracks (Mendez et al., 2005). This could be explained by the fact that in this case, cell suspension grafts were used instead of the solid tissue grafts in the previous three studies. Several factors, including vascularization, trophic factor support and graft–host interaction, may be different for single cell suspension grafts and tissue grafts (Leigh et al., 1994). In fact, cell suspension grafts have a lower number of immunogenic graft-derived blood vessels (transplantation antigens such as MHC I have one of their highest concentrations on endothelial cells and blood vessels) (Finsen et al., 1991) than solid tissue grafts that eventually supply the surviving cells. Hence, host-derived angiogenesis appear to be predominant in cell suspension grafts (Peschanski and Isacson, 1988; Geny et al., 1994; Leigh et al., 1994), which could make them less immunogenic to the host brain (Mendez et al., 2005).

Monitoring of the immune response after transplantation of other cell types has also been performed. The Phase II STEPS trial (NCT00206687, 2005), which evaluated safety and efficacy of a cell product consisting of cultured human retinal pigment epithelial cells on microcarriers called Spheramine^®^, made use of the immunohistochemical analysis of CD19 marker for B-cells, CD4 marker for helper T-cells, and CD8 marker for natural killers and cytotoxic T-cells in a *postmortem* case six months post-transplantation (Farag et al., 2009). This study, which failed to show clinical benefit and did not use immunosuppression, reported an inflammatory response that involved primarily macrophages with mild CD8-positive T-cell infiltration, in the absence of appreciable CD19 and CD4 immunoreactivity (Farag et al., 2009). Frequent infiltration by CD68-positive and other inflammatory cells in the needle tracts was observed (Farag et al., 2009).

More recently, results from the 4-years follow-up of the HSCfPD trial (NCT02780895, 2016) from Celavie Biosciences described an alternative method for monitoring immune responses. In this trial that used a cell-based product consisting of undifferentiated NPCs derived from human fetal brain tissue, named OK99, researchers showed a lack of an elicited immune response in one month CyA-immunosuppressed patient’s blood samples analyzed by flow cytometry for both NPC-specific antibodies and antibody-dependent cell-mediated cytotoxicity, and after CyA treatment withdrawal at six months after grafting (Madrazo et al., 2019).

Measurement of peripheral blood pro-inflammatory cytokines and chemokines has also been reported by one clinical trial (Use of Mesenchymal Stem Cells (MSCs) Differentiated Into Neural Stem Cells (NSCs) in People With Parkinson’s (PD). Full Text View – ClinicalTrials.gov., 2018) to monitor immune responses. Schiess et al. (2019) released the preliminary findings from a Phase I study which aimed to prove safety of intravenously-administered allogeneic hBM-MSCs in PD patients, in which levels of TNF-α, monocyte chemoattractant protein-1/C-C motif chemokine ligand 2 (MCP-1/CCL-2), macrophage-derived chemokine/C-C motif chemokine ligand 22 (MDC/CCL-22) and IL-9 were analyzed before and 3, 12 and 24 weeks after infusion (Table 1). An anti-inflammatory effect with a reduction of chemo-attractive molecules was reported following hMSC infusions for all the molecules monitored, which was also accompanied by significant increase in BDNF levels (Schiess et al., 2019).

One of the first studies providing a detailed evaluation of the immune response following bilateral implants of fWGE in HD patient’s caudate-putamen was a pilot study performed by Freeman et al. (2000). The immune response was evaluated in *postmortem* tissue 18 months after transplantation in one patient who died from cardiovascular disease. The analysis revealed considerable HLA-DR (MHC class II surface receptor, DR isotype) staining within host’s caudate and putamen while fewer HLA-DR positive cells were detected in the cell implant. There were no differences between CD4 and CD8 from host and graft and there was no perivascular cuffing, defined as regions with leukocyte aggregations. Upon the characterization of this patient immune response, the author’s concluded that minimal macrophage and T-cell immunoreactivity had been found (Freeman et al., 2000). *Postmortem* immunological studies of grafted tissue were complemented ten years later with the evaluation of the brains of three more HD patients (Cicchetti et al., 2011). Surprisingly, CD4, CD8 and HLA-DR markers were identified within the cell graft, thus indicating an activation of the immune response which was supported by the finding of a strong astrocytic response measured by glial fibrillary acidic protein (GFAP)-positive cells at the edges of the graft (glial scar). In addition, activated microglia were observed within the graft as well as in the surrounding area. Glutamate-mediated excitotoxicity was confirmed, apparently released from the activated microglia, which was postulated to contribute to promoting the degeneration of grafted cells. These studies (Cicchetti et al., 2009, 2014; Cisbani et al., 2013) concluded that the survival of the graft was severely compromised long-term, most likely due to an immunological response mediated by host atrophic astrocytes and activated microglia, the latest specifically targeting the neuronal components of the grafts and minimally affecting glia cells. The NEST-UK pilot study also reported to have measured inflammatory markers following transplantation. One of the NEST-UK cases (Rosser et al., 2002; Barker et al., 2013b) demonstrated microglial activation in *postmortem* samples, which were more dense around the grafts (Rosser et al., 2002; Barker et al., 2013b; Maxan et al., 2018).

The largest and most comprehensive study of the immune response in HD was done within the context of the multicentric intracerebral grafting in HD trial [MIG-HD; (NCT00190450, 2005)]. Fourteen months after the bilateral transplantation of fragments of fWGE in the striatum, one of the recipients exhibited a general worsening of the HD symptomatology and an acute weight loss. Graft rejection was confirmed as anti-HLA antibodies were detected in blood and CSF. Blood from twelve additional patients who had undergone identical surgery was also analyzed for anti-HLA antibodies, and found to be positive in four. This finding demonstrated alloimmunization towards donor antigens and called for a reconsideration of the immunosuppression given, pointing out the necessity of monitoring the immune response (Krystkowiak et al., 2007). In addition, the time at which anti-HLA appeared was highly variable as studied by Krebs et al. (2011), as it could happen during immunosuppression or shortly after in a completely unpredictable way.

There are clinical trials for safety assessment (NCT02728115, 2016), for analyzing dose-response (NCT03252535, 2017) and as an extension (NCT04219241, 2020), based on the intravenous administration of hMSC (CellAvita^TM^) to HD patients and whose approach include the measurement of the inflammatory markers such as IL-6, TNFα, IL-4 and IL-10 as well as the immune response by the measurement of lymphocytes CD4+ and CD8+ proliferation. However, there are no outcomes published about the immune response to date.

### Immunosuppression Regimen Given to Cell Therapy Approaches in PD and HD

Besides of the immune reaction evaluation, the administration of an immunosuppression regime also contributes to the outcome of the clinical trial. Table 1 and Figure 3B also summarize the immunosuppression therapy given in each pilot study or clinical trial for PD and HD using cell or fetal tissue therapy.

When transplanting fetal grafts for PD, immunosuppression therapy has been administered by many investigators although there were differences between groups: Olanow et al. (2003) administered Cyclosporine A (CyA) only for 6 months while the Lund group administered a triple immunotherapy (CyA, azathioprine and prednisolone) for longer periods (Lindvall et al., 1990; Wenning et al., 1997; Piccini et al., 1999). Freed et al. (2001) opted for not using any immunosuppression at all (Table 1). Based on the information available, it is not currently possible to determine an optimal immunosuppressive regime for striatal transplantation in the CNS. However, it is agreed by most groups working in this area that a period of immunosuppression is required and will involve at least one immunosuppressive agent, possibly for several years. Furthermore, it has been demonstrated that triple immunotherapy for a year after transplantation results in better DAergic cell survival, compared to no immunosuppression at all or monotherapy with CyA in PD patients (Barker et al., 2017).

The early pilot studies using allogeneic fWGE for HD treatment used either CyA alone (Šramka et al., 1992; Kopyov et al., 1998; Hauser et al., 2002a; Cicchetti et al., 2009), combined with prednisolone (Madrazo et al., 1995; Reuter et al., 2008) or used triple immunotherapy (Bachoud-Lévi et al., 2000b, 2006; Rosser et al., 2002). Where reported, clinical benefits seem to reflect the pattern observed in PD, being better when triple immunotherapy was administered (Figure 3B). It is worth mentioning that triple immunotherapy is not always applied consistently across studies. For instance, in the NEST-UK study the three agents (CyA, azathioprine and prednisolone) were all given post-transplantation, with patients weaned off prednisolone within six months. This is similar to what was done in the Florence study (where methylprednisolone was stopped after two weeks, maintaining azathioprine and CyA) or the MIG-HD trial (in which prednisolone was discontinued after 18 months). In contrast, the seminal Créteil pilot study discontinued cyclosporin A after 6 months, leaving the patients on the other two agents for 1 year. In the case of PSCs for PD patients, the first patient was transplanted with iPSC-derived DA producing neurons in 2018 (UMIN000033564, 2018). In this trial, patients are administered immunosuppression for 52 weeks post-transplantation, with tapering off over a 12 week period) (Stoddard-Bennett and Reijo Pera, 2019). However, this trial is currently suspended, with no result having yet being published.

MSCs seem to be more readily compatible with the host’s immune system as they have very low levels of MHC I and do not express MHC II molecules (Ryan et al., 2005; de Vasconcellos Machado et al., 2013). Although there is conflicting data, the majority of studies describe hMSCs as hypoimmunogenic cells that could escape recognition by alloreactive CD4-T cells (Ryan et al., 2005). In addition, there is evidence that MSCs may suppress the immune system and reduce inflammation by decreasing microglia and infiltrating leukocytes. Apparently, the lack of immune response against hMSC was maintained for allografts and xenografts, suggesting that hMSCs may represent a good candidate for immunomodulation following transplantation (Ryan et al., 2005). To our knowledge, none of the clinical trials of hMSCs to date included immunosuppression unless such information was omitted in the subsequent reports.

### Strategies to Overcome the Immune Response Following Intrastriatal Transplantation

**FIGURE 4:**
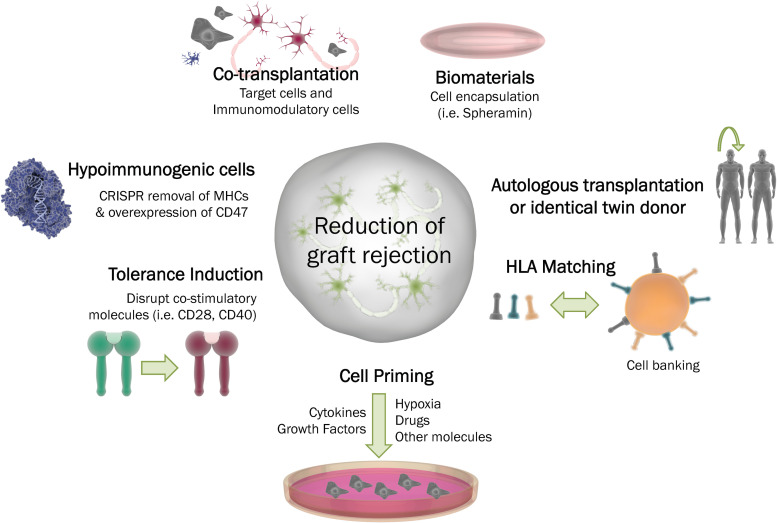
Strategies to overcome graft rejection. To diminish the immune system response following cell transplantation in the CNS, several strategies can be employed. Autologous transplantation or the procurement of tissue from an identical donor may be the best scenarios. Other possibilities include: cell priming through the addition of different factors to the cells (i.e., growth factors, drugs or cytokines); the induction of tolerance by deletion of co-stimutory molecules, the generation of hypoimmunogenic cells; the possibility of co-transplantation or encapsualtion of the cell graft into a biocompatible material. HLA: Human leukocyte antigen. MHC: Major histocompatibility complex.

Several strategies have been tested in order to overcome the immune response following intrastriatal transplantation (Figure 4). Indeed, the lowest immunogenic risk would be to graft autologous material or tissue from an identical donor twin. Currently, achieving these kinds of transplants for PD or HD patients in not straightforward, so a realistic alternative would be the selection of the best possible donor based on HLA compatibility with the host, which would still need to be accompanied by immunosuppressive drugs. In this regard, the generation of iPSCs cell banks would assure availability of HLA-matched tissue (Solomon et al., 2015; Morizane et al., 2017; Fan et al., 2020). However, this sort of approach is controversial, as a recent paper showed that MHC-matching neuronal grafts in a non-immunosuppressed non-human primate model of HD was insufficient to grant long-term survival of neuronal grafts. The main conclusion of this preclinical study is that a combination of MHC-matching graft with an adequate immunosuppression regime should be further investigated to obtain all the therapeutic potential that cell therapy carries (Aron Badin et al., 2019).

A common strategy to enhance immunomodulatory and overall therapeutic efficacy of hMSCs is by preconditioning them, or ‘priming’. Priming approaches are reviewed elsewhere (Ji et al., 2017; Noronha, Nc et al., 2019) and are mainly based on the application of growth factors and pro-inflammatory cytokines (IFN-γ, TNF-α, FGF-2, IL-1α and IL-1β), hypoxia, pharmacological drugs such as valproic acid, all-trans retinoic acid, and other molecules including lipopolysaccharide or cathelicidin.

Regarding the modulation of the cell potential to stimulate an immune reaction, two approaches have shown encouraging outcomes; hypoimmunogenic cells, and (closely related) tolerance induction. The recent idea of ‘hypoimmunogenic cells’, or ‘universal stem cells’ is receiving increasing attention as the regenerative medicine field is growing. Over the last year, Deuse et al. (2019) generated hiPSCs with deleted MHC-I and MHC-II genes and increased expression of CD47, a surface protein known as a “don’t eat me” signal (Brown and Frazier, 2001). Following differentiation into cardiac cell types, the newly generated cells were implanted in rodent models without any immunosuppression and survived long-term (Deuse et al., 2019; Shani and Hanna, 2019). Tolerance induction, on the other hand, relies on the use of more complex approaches that may include the blockade of co-stimulatory molecules that are crucial for T-cell activation, such as CD28-CD80/86 and CD40-40L (Liu et al., 2017).

Another interesting approach is the use of MSCs in combination with the target neuronal cells to immunomodulate the response upon grafting. Some studies have shown that MSCs can delay allograft rejection and generate a local immuno-privileged site in animal models, as well as preserve the functionality of the graft (Stewart et al., 2017; Vaithilingam et al., 2017; Razavi et al., 2018).

Finally, an approach that combines biomaterials and cell therapy has already reached clinical trial. The encapsulation of cells using biocompatible carriers has achieved substantial neuroprotective and neuroregenerative outcomes (Wong et al., 2014).

## Future Perspective: Standardization Through an Immunogenicity Testing Platform

To date, preclinical assessment of cell therapies has depended on transplantation into animal models, which is the only way in which to assess its effect on behavioral outcomes. However, despite the commonalities among mammalian immune systems, there are several differences between animals and humans at peripheral immune system (Colucci et al., 2002; Mestas and Hughes, 2004; Bailey et al., 2013; Zschaler et al., 2014; Haley, 2017) and CNS (Zhang et al., 2016; Galatro et al., 2017; Gosselin et al., 2017; Masuda et al., 2019), thus introducing a degree of uncertainty in terms of understanding the immune responses to a cell therapy product that may occur in humans.

During the administration of a given cell therapy, the process of surgical intervention inevitably generates tissue damage in the brain. The molecular events taking place in a human healthy brain during such procedure are not fully understood, and are even less clear in diseased brains that are already subjected to chronic or acute neuroinflammation, as in PD and HD. This is one of the main challenges for cell therapy. The second major challenge is that although the host response has been assessed to some extent in several clinical trials (Table 1), the graft response to the host resident immune cells in the brain parenchyma and those migrating from the periphery have not been fully considered. As described in this review, the immune response to the graft is highly variable and unpredictable (Krystkowiak et al., 2007; Krebs et al., 2011). In this sense, a possible strategy for systematically testing the immunogenicity of cell therapy and other ATMPs is an *in vitro* human-based platform. A well-designed platform could give valuable information about molecular and cellular aspects, contributing to the standardization of pre-clinical safety assessment of cell therapies. For these reasons, we highlight some features to be considered in the development of a neuro-immunogenicity testing platform.

The immunogenic response in the brain depends either on the host’s neuroimmune system and/or on its peripheral immune system response, especially if cells are administered intravenously, as for hMSCs. From this perspective, we envision a two-way platform for testing the effects of potential cell therapies on the peripheral immune system and/or on the neuroinmmune system response. To mimic the physiological communication of these two compartments, the presence of a human relevant BBB models is a minimum requirement at the interface. In fact, the BBB is not only a barrier, but it is also associated to major neuroimmune functions (Erickson et al., 2020).

**FIGURE 5:**
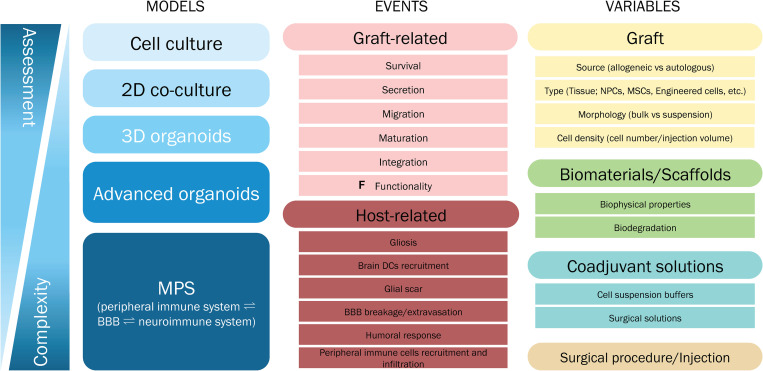
Immunogenicity testing scenario. A perfect model to assess the immunogenicity of cell transplantation therapy does not exist due to the complexity of the system. A tiered approach could provide an approximation depending on the cell therapy aspects of interest that we want to test. The immunogenicity testing can be focused on a particular element of the system, which will determine the most relevant model to work with. Some specific variables that need testing will require advanced and more complex models. As advanced organoids, we envision homogeneously cultured organoids including microglial cells, DCs, vascularization and functional BBB. MPS = microphysiological system.

In our view, the testing platform should be designed as integrated modules. The concept of modularity permits starting with simple testing, saving time and resources, with a tiered approach to slowly upgrade the complexity level (Figure 5). If a graft candidate is already stimulating microglia in a 2D culture, we may assume that the graft is inducing a certain level of immunogenicity. It is also true that the presence of astrocytes and neurons in the culture could dial down the microglial activation (Biber et al., 2007). The tiered approach for immunogenicity testing would allow the dissection of molecular events in each condition tested.

In the case of HD, the immunogenicity test must be performed in cellular models possessing the HTT mutation, which is achievable by using hiPSCs or genetically engineered cells (Csobonyeiova et al., 2020) as starting material to derive neurons (Conforti et al., 2018; Comella-Bolla et al., 2020), oligodendrocytes (Li et al., 2016a; Ehrlich et al., 2017; Garcia et al., 2019), microglia (Sabogal-Guáqueta et al., 2020), DCs (Sachamitr et al., 2018), endothelial cells (Karow et al., 2012), and pericytes (Karow et al., 2012), to consistently model human brain parenchyma and BBB (Raimondi et al., 2020). A similar strategy could be applied to PD using known genetic variants (Garcia et al., 2019) as a relevant cellular phenotype is currently more difficult to achieve for sporadic PD (Sancho-Balsells et al., 2020). Human iPSCs can be also used to ensure the presence of the multiple genetic polymorphism observed in PD (Ferrari et al., 2020). However, environmental triggers would be missing, and in PD microglia can either be activated by degenerating DA neurons or can be already activated through the neurodegeneration exacerbating microglial neurotoxic responses (Fellner et al., 2011). Furthermore, the BBB is reported to be dysfunctional in PD and HD patients (Gray and Woulfe, 2015) so this is also an important feature to model into a future immunogenicity platform connecting the CNS immune system to the periphery. The initial approach should test the cell graft, including types of cells and donor source/origin, scaffolding materials, and coadjuvant solutions, which can be performed in 2D co-culture models composed of human iPSC-derived microglia, astrocytes and neurons. A more complex approach could study the impact of various graft-related morphological features on the immunogenic potential, such as bulk tissue versus suspension, cell density, and surgical materials or scaffolds. This will require homogenous culture of 3D models such as brain organoids (Lancaster et al., 2013; Conforti et al., 2018; Qian et al., 2018). The 3D models will approximate to the relevant biophysical environment by modeling the surgical procedure by microinjection of an in-scale graft. A protocol to generate striatal organoids has been described, although microglia and astrocytes were missing (Adil et al., 2018).

To mimic *in vitro* the surgical procedure, there are some challenges to tackle; mainly allometric scaling and neuronal/glial cell density ratio in the human brain tissue. For practical reasons the spatio-temporal phenotypic differences of astrocytes and microglia (de Haas et al., 2008; Koning et al., 2009) cannot be taken into account as first approximation, although it is possible that glial migration into the 3D models will trigger functional differences due to the regionalization process (Qian et al., 2018). In any case, glial regional cell density should be considered as a controlled variable to generate relevant 3D striatal models. In fact, microglia and astrocytes show different distributions depending on the brain region (Lawson et al., 1990), and the number of glial cells may vary in HD (Palpagama et al., 2019) and PD models (Fellner et al., 2011).

The ideal solution for any research platform would be to have high-throughput and high content analysis, but this is not feasible in a field in which technological advances are at an early stage. Hence, the productivity and high-content analysis are inversely proportional. To choose what to prioritize depends on the specific clinical focus. In testing cellular immunogenic responses of a graft, a high-content analysis would be of interest to understand the molecular cascades and activated pathways in depth. When testing surgical materials, solutions and scaffolds, high-throughput analysis seems more appropriate as it allows for rapid screening of immunogenic properties at single-cell levels. However, to test complex variables such as the biophysical properties or integration into the brain parenchyma, advanced models must be employed to ensure relevant data that can be translated into increased clinical efficacy and safety.

### Biological Endpoints

The first target of analysis would be to profile the immune response. However, the neuroimmune system is a highly regulated and complex network and the immune response is not so simple to evaluate. In fact, microglial activation has not been straight-forward to determine, since many microglial functions can damage the graft at different stages through a variety of mechanisms, such as ROS production, phagocytosis, cytokine production and synaptic pruning, etc. (Bachiller et al., 2018). In Figure 5, we present some events to establish the biological endpoints, depending on the different stages of the cell transplantation and integration into the striatum. In any case, the minimal biological endpoints should include the assessment graft survival and cell viability, gliosis, neuronal synaptic plasticity, and cytokine and chemokine production. These endpoints can be easily measured in any neuroscience research lab with standard methods of analysis from 2D co-culture models onwards.

It is also highly relevant to distinguish between the acute and chronic immune response. In fact, peripheral and neuroimmune systems communicate in a highly regulated manner and this must be studied in complex models integrating both compartments and cellular components. However, modeling long term immunogenic responses could be technically challenging in microphysiological systems integrating more than one compartment, each with different cells and conditions. Furthermore, there is still a lack of knowledge about the events leading to chronic immune rejection in the brain. The use of an *in vitro* immunogenicity platform, organized as modules that mimic different physiological compartments, connecting peripheral and neuroimmune system through the BBB, would also enable biomarker discovery. The correlation between the neuroimmune activity with peripheral measurable biomarkers is of great importance for monitoring patient recipient state, and has the potential to substantially improve the follow-up of grafts in HD and PD patients following translation to the clinic. However, to date fully functional immunogenicity platforms have not been applied to the development and preclinical testing of cell therapies in neurodegenerative diseases.

## Conclusion

Several efforts have been undertaken to successfully apply cell therapy in neurodegenerative diseases such as HD and PD. To date this approach remains experimental and, despite being highly promising as disease-modifying therapy, relatively few clinical trials have been undertaken and most of these have been small and under-powered to assess efficacy. One of the challenges when considering cell therapy for brain diseases is the immune response that occurs in the CNS. The immune response can compromise the survival of grafted cells, impairing their integration and therefore, their functionality. Most clinical assays in the field are not accompanied by clear and feasible guidelines for monitoring the immune response (Table 1). In addition, the immunosuppressive treatments used to date have been highly variable (Figure 3B, and the associated adverse effects not always comprehensively reported. For this reason, we have also summarized the neuro- and inflammatory markers that could be used to shape the guidelines of future transplants in the CNS.

In recent years, *in vitro* strategies have been developed to evaluate the potential immunogenicity of cell therapy, and these have the potential to address immunological issues that cannot be readily addressed in animal models. Funding agencies and neuroscience community should look to this kind of platform as a priority to improve and standardize preclinical studies for the development of cell therapies. Good well standardized *in vitro* models may provide access to understanding some mechanism that are difficult to assess in *in vivo* models.

## Author Contributions

All authors contributed to the writing and revision of the text.

## Conflict of Interest

MS is employed as CEO by the company SCIENCE&STRATEGY SL. The remaining authors declare that the research was conducted in the absence of any commercial or financial relationships that could be construed as a potential conflict of interest. The handling editor declared a shared affiliation, though no other collaboration, with several of the authors CS-M, UP, FM-R, and JC at the time of review.
